# Acceptability and feasibility of assisted telepsychiatry in routine healthcare settings in India: a qualitative study

**DOI:** 10.1093/oodh/oqad016

**Published:** 2023-10-19

**Authors:** Abhijit Nadkarni, Ankur Garg, Ravindra Agrawal, Seema Sambari, Kedar Mirchandani, Richard Velleman, Devika Gupta, Urvita Bhatia, Godwin Fernandes, Ethel D’souza, Akshada Amonkar, Anil Rane

**Affiliations:** Centre for Global Mental Health, Department of Population Health, London School of Hygiene & Tropical Medicine, Keppel Street, London WC1E 7HT, UK; Addictions and Related Research Group, Sangath, Porvorim, Goa 403501, India; Addictions and Related Research Group, Sangath, Porvorim, Goa 403501, India; Addictions and Related Research Group, Sangath, Porvorim, Goa 403501, India; Addictions and Related Research Group, Sangath, Porvorim, Goa 403501, India; Addictions and Related Research Group, Sangath, Porvorim, Goa 403501, India; Addictions and Related Research Group, Sangath, Porvorim, Goa 403501, India; Department of Psychology, University of Bath, Claverton Down, Bath BA2 7AY, UK; Addictions and Related Research Group, Sangath, Porvorim, Goa 403501, India; Addictions and Related Research Group, Sangath, Porvorim, Goa 403501, India; Addictions and Related Research Group, Sangath, Porvorim, Goa 403501, India; Addictions and Related Research Group, Sangath, Porvorim, Goa 403501, India; Addictions and Related Research Group, Sangath, Porvorim, Goa 403501, India; Institute of Psychiatry & Human Behaviour , Bambolim, Goa 403108, India

**Keywords:** telepsychiatry, primary healthcare, lay-counsellor, India, qualitative study, acceptability, feasibility

## Abstract

Technology-enabled interventions are often recommended to overcome geographical barriers to access and inequitable distribution of mental healthcare workers. The aim of this study was to examine the acceptability and feasibility of an assisted telepsychiatry model implemented in primary care settings in India. In-depth interviews were conducted with patients who received telepsychiatry consultations. Data were collected about domains such as experience with communicating with psychiatrists over a video call and feasibility of accessing services. Data were analysed using a thematic analysis approach. Patients recognized that technology enabled them to access treatment and appreciated its contribution to the improvement in their mental health condition. They reported that the telepsychiatry experience was comparable to face-to-face consultations. They had a positive experience of facilitation by counsellors and found treatment delivery in primary care non-stigmatizing. While some adapted easily to the technology platform because of increased access to technology in their daily lives, others struggled to communicate over a screen. For some, availability of care closer to their homes was convenient; for others, even the little travel involved posed a financial burden. In some cases, the internet connectivity was poor and interfered with the video calls. Patients believed that scale could be achieved through adoption of this model by the public sector, collaboration with civil society, enhanced demand generation strategies and leveraging platforms beyond health systems. Assisted telepsychiatry integrated in routine healthcare settings has the potential to make scarce specialist mental health services accessible in low resource settings by overcoming geographical and logistical barriers.

## Teaser

Lay summary: People with mental health problems have inadequate access to appropriate care across the world. Technological solutions can be an important enabler to overcome barriers related to geography and inequitable distribution of mental healthcare workers. We implemented an assisted telepsychiatry program in primary care settings in India and conducted interviews with the beneficiaries to understand their experience of communicating with psychiatrists over a video call**.** On the one hand, patients recognized that technology enabled them to access treatment, found the telepsychiatry experience comparable to face-to-face consultations, appreciated its contribution to the improvement in their mental health condition and found treatment delivery in primary care non-stigmatizing. On the other hand, some patients struggled to communicate over a screen and found that even the little travel involved in getting to the primary health centre was financially burdensome. A program such as this has the potential to make scarce specialist mental health services accessible in low-resource settings by overcoming several barriers to care.

## INTRODUCTION

Between 1990 and 2019, the global number of disability-adjusted life years (DALYs) due to mental disorders increased from 80·8 million to 125·3 million, and the proportion of global DALYs attributed to mental illnesses increased from 3.1% to 4.9% [1]. Around a third of these global DALYs is contributed by two countries (66 million DALYs), one of which is India, and this is much greater than all developed countries combined (50 million DALYs) [2]. One in seven Indians (i.e. 197.3 million individuals) were affected by mental illnesses of varying severity in 2017 [3].

There is substantial evidence supporting the cost-effectiveness of treatments for a range of mental illnesses such as depression or schizophrenia, including evidence from a number of low- and middle-income countries (LMICs) [4]. Despite that, access to care for people with mental illnesses remains limited—globally, the treatment gap for people with mental illnesses is more than 50% and is as high as 90% in the least resourced countries [5]. For example, in India, only 2 out of 10 individuals with mental illnesses receive any formal mental healthcare—a treatment gap of 84.5% [6].

Globally, there are several demand-side (e.g. stigma, lack of awareness) and supply-side (e.g. shortage of skilled workers, inequitable distribution of limited resources, geographic distances) barriers that contribute to poor access to mental healthcare. One key reason for such a high treatment gap, especially in LMICs, is the shortage of skilled mental healthcare workers. For example, in countries such as Chad, Liberia and Rwanda, there are only one or two psychiatrists serving the entire country [7]. India has 0.75 psychiatrists per 100 000 population, much lower than a very conservative estimate of six psychiatrists per 100 000 population in high-income countries [8]. Additionally, the scarce resources are distributed inequitably and inefficiently within LMICs—substantial investments have been made in large mental health institutions, and mental health professionals tend to provide services in and around the largest cities [7]. This results in significant geographical barriers to access, especially to those with the highest need such as the poor, those who are least educated, women, young people and rural inhabitants [7]. Faced with such extreme workforce shortages magnified by geographical barriers to access, traditional approaches such as greater investment in psychiatric training programmes and even innovative strategies such as task sharing with non-specialist health workers will not be sufficient to close this gap [9].

Several reviews have synthesized evidence from studies of telepsychiatry (use of a live video and audio connection for a clinical session), primarily from high-income countries, and concluded that it (a) is an innovative strategy to improve access to mental healthcare and ultimately reduce the treatment gap [10]; (b) is associated with high levels of patient satisfaction, diagnostic reliability and improved clinical outcomes [11]; and (c) has comparable accuracy of assessment and patient satisfaction compared to face-to-face care [12]. This makes it an especially attractive alternative to traditional face-to-face care, especially in settings with limited availability and inequitable distribution of specialist providers, or geographical challenges to accessing care [13]. The evidence for the use of telepsychiatry in LMICs is emerging and needs further exploration [14, 15], particularly its integration into routine healthcare settings to increase accessibility.

The IMPACT (IMproving Access through Tele-psychiatry) telepsychiatry program was developed through a systematic research process of examining the existing global evidence on telepsychiatry services and in-depth interviews with a range of stakeholders (including psychiatrists, patients and caregivers) to understand the process, synergies with public sector primary healthcare systems and potential limitations of providing telepsychiatry in the community. The resulting contextually appropriate ‘assisted telepsychiatry’ program was integrated into routine clinical settings for proof-of-concept testing in Goa, India [16]. Eligible patients attended tele-consultation sessions with the psychiatrist, delivered through a customized online platform. The sessions were facilitated (‘assisted’) by a lay counsellor to support the use of the online software.

Of the 126 participants who entered treatment in IMPACT, 58 (46%) received only tele-consultation from the psychiatrist, and the rest received both tele-consultation from a psychiatrist and counselling from a lay counsellor. A total of 626 sessions were conducted with patients comprising both assisted tele-consultation from psychiatrists (64%) and face-to-face counselling from lay counsellors (36%). Overall, 102 (81% of those who entered treatment) participants had a planned discharge from the treatment. There was a statistically significant reduction in both psychological distress and disability between baseline and follow-up.

The aim of this paper is to supplement the quantitative findings summarized above with a description of the acceptability to the patients and feasibility of delivering the IMPACT program, which we developed and tested using a systematic intervention development process.

## MATERIALS AND METHODS

### Study design

#### Qualitative study using in-depth interviews

The program was implemented between December 2017 and March 2020. The in-depth interviews (IDIs) were conducted between April 2018 and March 2020 with patients who had received at least one telepsychiatry session and already been discharged from care.

### Setting

Goa (population 1.4 million), on the west coast of India. Mental healthcare in Goa is provided by private healthcare providers and by the public sector through a tertiary care teaching hospital, two district hospitals and the District Mental Health Program. Private mental healthcare is expensive and largely restricted to urban areas, and the rural areas are particularly underserved. Access to public sector specialist mental health services requires residents of the study areas to travel substantial distances using infrequent public transport. IMPACT aimed to reduce the geographical barriers to access by providing free specialist services in the public sector health facilities closer to where people lived. Participants for IMPACT were recruited (a) directly from a rural community through self-referral in response to dissemination posters/flyers in the community and (b) universal screening in public sector health services—the outpatient departments of one community health centre, two primary health centres and one general hospital.

### Sample

IMPACT provided services to 126 adults (⩾18 years). Participants were screened using the Global Health Questionnaire (GHQ-12) [17] and Alcohol Use Disorders Identification Test (AUDIT) [18]. Both these tools have been validated in India [19, 20] and extensively used in the study setting [21, 22]. Those who screened positive on these tools or were identified as having a possible mental disorder by the lay counsellor (based on their clinical judgement) were then assessed by the psychiatrist to make a final diagnosis [16]. We excluded patients (a) requiring immediate medical attention, (b) not planning to reside in the study catchment area for the next 6 months or (c) unable to speak in either English or one of the three vernacular languages—Hindi, Konkani or Marathi.

Out of the 126 patients who received services from IMPACT, we purposively selected 20 for the IDIs to achieve maximum variability on key socio-demographic variables (age, gender, education, employment status). As the focus of this nested sub-study was on acceptability and feasibility of the delivery platform, and not the content of the intervention, we did not use diagnosis as one of the parameters for selection of participants. All the participants that we approached for the IDIs consented to participate in the study.

### Intervention

The tele-consultation sessions were provided by one of two psychiatrists with medical degrees, specialist psychiatric training and more than 5 years of experience of clinical practice. They were assisted by a group of four lay counsellors who are essentially non-specialist workers who were educated to at least senior secondary level and had subsequently been trained to deliver basic counselling and evidence-based psychological treatment. Eligible and consenting patients were provided with the first assisted tele-consultation session of 20–30-minute duration with the psychiatrist delivered through the customized online platform. The lay counsellor would first conduct a brief clinical assessment of the patient before the session and would then brief the psychiatrist. The sessions were facilitated by the lay counsellor who would sit in with the patient to support them in the use of online software. The treatment plan, including the number and duration of sessions, was decided by the psychiatrist in consultation with the patient and the lay counsellor. If medications were indicated, then a prescription to purchase the medications would be shared by the psychiatrist through the online platform and a printed copy was given to the patient by the lay counsellor. If recommended by the psychiatrist, the lay counsellor provided face-to-face counselling services to the patients, which included client-centred supportive counselling and a contextually appropriate evidence-based psychological treatment package for either depression (Healthy Activity Program - HAP) [23] or harmful drinking (Counselling for Alcohol Problems - CAP) [24]. The lay counsellors also provided visits to patients’ homes to conduct sessions in situations where patients were unable to attend the facilities due to difficulties such as childcare or caring for sick persons at home.

The telepsychiatry platform was based on an existing proprietary software. We selected the vendor because of his willingness to promptly adapt the interface to meet the specific requirements of our program. The features of the telepsychiatry platform included appointment scheduling, video conferencing, history-taking format, generating and sharing of notes and files, medicine prescription printing and session timer. The psychiatrist’s dashboard displayed a summary of upcoming sessions and completed sessions with the clinical notes made during previous sessions.

### Data

The interview guide was designed to achieve the study objectives related to acceptability (perception amongst implementation stakeholders that a service is agreeable, palatable or satisfactory) and feasibility (extent to which an innovation can be successfully used or carried out within a given setting) [25]. The patients were asked about their experience of the telepsychiatry portal (e.g. quality of audio-visuals), of consulting a psychiatrist via a video call, of the lay counsellor’s role in aiding the tele-consultation and challenges faced in accessing this treatment. Five trained research workers (two males and three females) with a Bachelor’s or Master’s degree conducted the IDIs in the vernacular language. After engaging with the patients, they were interviewed face-to-face and privately at their homes, at the clinic or in neutral spaces such as a temple or park as per their convenience. Quality of data was monitored on an ongoing basis through robust mechanisms such as the following: all interviews were audio-recorded and lasted between 30 and 45 minutes. The interview content was examined through regular feedback from the supervisor and peers by listening to the audio recording of the interviews. Feedback was provided on the flow of the interview; probing techniques, asking the right questions and suggestions for improvement were provided to the interviewer. Group supervision ensured peer learning through the feedback given to the researchers on a regular basis. All audio-recorded interviews were translated and transcribed into English. Data collection was discontinued once we reached theoretical saturation and were reasonably assured that further data collection would only serve to confirm emerging themes and conclusions.

### Analysis

Data were analysed using the thematic analysis approach with the NVivo software. Our epistemological position recognizes that some aspects of acceptability and feasibility of an intervention are not directly observable and have to be inferred through an examination of the recipients’ subjective experiences. The transcripts of the interview were read by two individual researchers (A.G. and S.S.) for familiarization of the data. Using a deductive approach, the researchers then generated preliminary codes and grouped them into themes and sub-themes based on the original research questions and sections in the topic guide. The reliability of the coding system was tested by carrying out blinded, double coding of the first 10 transcripts to reach a consensus on the preliminary codes and themes. The broad coding themes included (a) acceptability of telepsychiatry; (b) feasibility of telepsychiatry; and (c) recommendations for changes. The researchers developed a comprehensive codebook from a set of 10 transcripts that was reviewed by A.N. and U.B. to finalize the initial codebook. A.G. and S.S. coded the remaining transcripts and generated higher order themes and sub-themes. Finally, a narrative interpretation of the themes was developed, and illustrative quotes were selected. Both the coders had direct experience of the study setting and included a field worker (S.S.) and project coordinator (A.G.), neither of whom were directly involved with the intervention delivery. The wider research team included senior-, mid- and early-career researchers who contributed to IMPACT in various capacities. These perspectives facilitated the interpretation of the data accounts as related to a priori theoretical and empirical foundations of complex intervention development and testing in the study settings.

### Ethical considerations

Ethical approval for the study was obtained from the Institutional Review Board of the implementing organization. Written informed consent was obtained from all participants. All participants were already receiving care for their mental health problems from IMPACT when they participated in these IDIs.

## RESULTS

Table 1 summarizes the sociodemographic profile of the 20 participants in this nested qualitative study and compares it to the overall sample of participants who received treatment (*n* = 126).

**Table 1:** Sociodemographic profile of participants

	**Qualitative study participants *n* = 20 N (%)**	**Participants in IMPACT program *n* = 126 N (%)**
**Mean age in years (SD); range**	49.8 (11.6); 28–74 years	51.8 (14.1); 18–93 years
**Gender**		
Female	15 (75)	82 (65.1)
Male	5 (25)	44 (34.9)
**Education status**		
No formal schooling	4 (20)	29 (23.0)
Completed primary	3 (15)	26 (20.6)
Completed secondary	7 (35)	48 (38.1)
Completed higher Secondary	4 (20)	10 (7.9)
Graduate	2 (10)	12 (9.5)
Postgraduate	0 (0)	1 (0.8)
**Employment status**		
Employed	4 (20)	44 (34.9)
Retired	2 (10)	15 (11.9)
Homemaker	14 (70)	65 (51.6)
Unemployed	0 (0)	1 (0.8)
Student	0 (0)	1 (0.8)
**Experience of technology**		
Mobile phone	18 (94.7)	101 (80.8)
Smartphone	5 (26.3)	36 (28.8)
Computer	1 (5.3)	16 (12.8)
Internet usage	4 (21.0)	28 (22.4)
**Medications prescribed**	10 (50.0)	73 (57.9)
**Treatment modality**		
Consultation with psychiatrist	7 (35.0)	58 (46.0)
Consultation with psychiatrist and counselling by lay counsellor	13 (65.0)	66 (54.0)
**Treatment status**		
Planned discharge	17 (85.0)	102 (81.0)
Unplanned discharge	3 (15.0)	24 (19.1)
**Mean number of sessions (SD)**	5.10 (3.08)	4.96 (3.73)
**Mean duration of sessions (SD)**	29.85 (20.07)	36.80 (35.51)

The following sections describe the factors that influence the acceptability and feasibility of IMPACT and recommendations to facilitate scale-up of telepsychiatry programs.

### Acceptability

Patients recognized that technology enabled them to access mental health treatments and provided them with the opportunity to interact with the psychiatrist, which was not present earlier.

‘*So, using technology, we can sit here* (primary care clinic close to their home) *and get our problems solved from doctors who are far. It is a very good system*’. (M, 57 years).

They also reported that the experience of the consultation received through the online platform was comparable to face-to-face consultations with other doctors.

‘*I felt like the doctor was sitting in front of me. I felt good. I felt that there will be some way out from this* (mental health problem)’. (M, 43 years).

However, some patients were hesitant to communicate with the doctor over a screen due to the unfamiliarity with the medium and felt awkward. A few patients reported facing challenges with technology, and some of them preferred in-person doctors over a remote clinician.

‘*She* (counsellor) *started the laptop and said that now the doctor will talk with me. That time I felt like, how will I talk. I could see the doctor with my eyes, but how should I respond. This is what I felt*’. (F, 55 years).

‘*First, I thought that he* (psychiatrist) *will be present in front of me* (in person)*. One can speak properly with him like that. This way* (over the laptop) *it becomes a little difficult... if you have to tell him something*’. (F, 54 years).

Spending time consulting the psychiatrist through the telepsychiatry portal resulted in a positive experience for the patients. They reported satisfaction and appreciated its contribution to the improvement of their mental health concerns. Patients also mentioned how the treatment benefited them (e.g. receiving appropriate advice) and resulted in positive changes in their life including better emotional health and adoption of healthy behaviours.

‘*Previously I would not spare time for myself as such. I would not go for a walk or to do other things. I would not think about my life or anything about my health. If there was work to do, I would keep on doing the work. Not anymore. Now I leave the work aside and think about what is to be done for my health and I do it*’. (F, 46 years).

‘*Improve in the sense if somebody* (Psychiatrist) *is giving so much time. This was my point that I told you also in the beginning that in today’s life nobody has time for anybody*’. (F, 40 years). Similarly, patients reported their experience with the counsellors facilitating the telepsychiatry sessions as predominantly positive, with them being described as cooperative, encouraging, trustworthy and courteous.

Increased access to technology in people’s daily lives and consequent ease with using technology was key in reducing any potential discomfort (resulting from unfamiliarity with digital devices) with telepsychiatry.

‘*Now even the elderly know how to use a mobile phone. So, they do not have an issue with talking* (using videoconferencing)*. They are satisfied just by the fact that the doctor is talking to them. People under 50 years have all become tech savvy. They will not have any issues with this* (telepsychiatry)*. But even for those above 50 years they won’t have much issues as they do have some idea* (experience of technology)’. (F, 40 years).

Other facilitating factors included the delivery of treatment in primary care, home visits if the patients could not travel to the primary care clinics and free treatment. They reported that availability of psychiatry and counselling services for ‘mental distress’ (psychological and emotional problems) was helpful as it was not perceived as ‘mental illness’ (abnormal behaviour) that is stigmatized and discouraged help-seeking.

‘*If we come here* (Primary Health Centre) *to seek help, then it is not that we have a mental illness. People consider “mental” means being “mad”... it is not like that. There is a lot of difference between going to the “mental hospital” for treatment and seeking help for mental issues (*distress*) here’*. (M, 74 years).

On the other hand, some patients experienced challenges in taking up offers of treatment because of stigma around mental health issues and how the word ‘mental’ is associated with being ‘mad’.

‘*What happens is that one gets scared even to ask* (for help) *from anyone. When you say “mental,” they take it to be literally mad* (vernacular interpretation for psychoses)*. Mental health problems are many (*and not only “madness”*). “Mental” doesn’t mean one is mad*’. (M, 74 years).

Some patients reported that there was a lack of support from their family in accessing the treatment and there were concerns around the gender of the counsellor (especially if the counsellor undertook a home visit).

‘*I do not want a man (*male counsellor visiting*). People shouldn’t say anything. It is OK to visit according to me. But what happens is, what the other person (*neighbours*) seeing this will say? It can be misinterpreted, right? You can also come like this... in a month or in fifteen days. But I do not want a male person* (male counsellor) *over here*’. (F, 54 years).

Although patients were given the option of home visits for receiving treatment, not all patients accepted the offer and chose to visit the health facility instead. This might have been due to perceived stigma pertaining to the curiosity from the neighbours, amongst other reasons.

‘*Those ladies who gathered at my house* (curious neighbours) *when he (*counsellor*) last came to my house to enquire about me? Instead of people (*neighbours*) talking about this, it is better that I make the effort to visit the PHC for the session’.* (F, 65 years).

Some patients discontinued the treatment midway and did not complete all the sessions as they felt better with a few sessions and did not feel the need to continue the treatment. Others discontinued treatment as they did not perceive it to be potentially helpful. One such patient said ‘*Not that I wasn’t called* (reminded about appointment)*. I told Madam* (counsellor) *that I will not need it* (treatment)*. I heard what you had to say. But I am happy with my way of handling things. So, I am handling myself*’. (M, 57 years).

### Feasibility

The primary reported facilitators to accessing the treatment were the ‘shorter distance’ patients needed to travel, as the treatment was available in health centres close to their homes and the ‘reduced amount of time’ it took them, as compared to travelling 45–60 minutes to get to tertiary care facilities (which are typically situated in urban areas). This was further enhanced by the benefits of assistance (by the counsellor), i.e. ease of appointment scheduling and the option of home visits. Elaborating on the benefit of home visits to allow access to telepsychiatry services, a patient said ‘*She* (counsellor) *told me to speak* (using the IMPACT portal)*. She had come to my home with the laptop. Because it was at home, I wasn’t that scared. If there were many people, then I would have got scared*’. (F, 60 years).

However, for some patients, travelling for the treatment (even if it was to the PHC) posed its own challenges. Some patients reported financial barriers (travel costs) and difficulties due to poorly connected areas and infrequent public transport. Dependence on family members for transportation was another barrier for patients, especially women, as they were dependent on a male member of their family to take them even to the nearest health facility.

Quality of technology such as good audio and video quality during the sessions were highlighted as positive characteristics of the treatment*. ‘I was able to hear well from their sound system. And I was able to reply to all the questions*’. (M, 74 years). However, this was not always the case, and sometimes, the audio and video quality of the sessions was sub-optimal—some patients did report disturbances during the session on the online platform due to poor and inconsistent internet connectivity in some instances.

While all appreciated the free consultations, a key challenge was availability and cost of prescribed medications. IMPACT did not provide free medications to the patients, and hence, patients had to depend on the public healthcare system or private pharmacies to purchase medications out-of-pocket when they were not available for free in the public healthcare system. A few patients, who were prescribed medications, reported challenges in procuring medicines due to non-availability, dependence on family members to pay for the medicines, administrative challenges in getting medicines in the public sector and their own financial limitations. Commenting on the bureaucratic process to get medications, one patient said ‘*I was already stressed, and then those people from the hospital, those nurses in the pharmacy, would repeatedly send me back and forth to the doctor to get things in writing. So that was something that I did not like*’. (F, 45 years).

### Recommendations for scaling up

When specifically asked about any changes that they would suggest to make this a better programme, the participants made the following suggestions:

#### Adoption by existing public health services

Patients recommended that telepsychiatry should be proposed to the government so that such a service could be made available at all healthcare facilities free of cost. One patient said ‘*But hope and pray to God, I am telling the truth... I will pray to God that this becomes a government aided program’.* (F, 40 years).

#### Collaboration with other organizations

Some patients suggested that collaboration between non-governmental organizations, for instance, would be necessary to extend the reach of such a program. One patient said ‘*Or else you can do one thing... now there are NGOs... which work selflessly. You can connect with them and tell them that this is the problem and ask them if they can help. If they are able to do it, then the problem gets solved*’. (M, 57 years).

#### Demand generation

Increasing awareness about the telepsychiatry services using methods that are accessible was suggested as a strategy to increase the reach of the services. One patient said ‘*You have to do awareness* (in the community)*. Now even this board* (poster about IMPACT) *that is put up, out of 10, one person will read this. Also, everybody who reads it, will not understand everything written on it*’. (M, 74 years).

#### Alternative sites

General healthcare facilities, even though less stigmatized than conventional mental health service facilities, still had stigma attached to them when it came to people seeking help for mental health problems. Patients suggested that telepsychiatry should be delivered at other places such as a separate office (not linked to the hospital) or any other neutral space for better acceptance and uptake of treatment.

‘*You must be having an office outside. People will visit more over there. When it comes to hospital, people feel that it is for “mental” patients. If you have your office outside, then people will come there with family, and society will not be curious about why you are going there*’. (F, 45 years).

## DISCUSSION

Through an uncontrolled evaluation of the IMPACT telepsychiatry program, we were able to demonstrate that treatment of mental illnesses through a telepsychiatry platform was feasible and was associated with improved clinical outcomes [16]. Through this qualitative study nested in the aforementioned quantitative study, we were able to corroborate some of the findings. The experiences narrated by these patients who received services through IMPACT indicate that despite certain systemic challenges, telepsychiatry is a viable option for leveraging technology to increase access to mental healthcare. This is especially relevant in settings constrained by limited availability and inequitable distribution of specialist mental healthcare professionals. Such evidence is critical as the translation of effective interventions into clinical practice requires patients to be willing to consider the intervention and be satisfied with it and policy makers to consider investing in it based on its feasibility.

The disparities in the availability of mental health services between and within countries requires innovative strategies such as the use of technology to increase access to care. As demonstrated in our work, telepsychiatry is one such solution that finds good acceptability amongst the eventual beneficiaries. However, in many countries, the digital divide ensures that people with low income, receiving public benefits and hailing from cultural and linguistically diverse communities may not have access to even ‘basic’ digital technology [26]. Our model of assisted telepsychiatry addresses this barrier wherein trained lay counsellors facilitate scheduling and setting up telepsychiatry consultation either in the primary healthcare facility or by conducting a home visit. The advantages of such an ‘assisted’ model will be further amplified in settings where there is low digital literacy and/or limited ownership of compatible digital devices, both of which can further exacerbate the inequitable access to mental healthcare.

While the use of telehealth services has increased exponentially since the COVID-19 pandemic [27], this increase has been mainly in urban populations with well-established access to technology. However, the penetration and uptake of these services is limited in rural populations, elderly and those with limited digital literacy and poor bandwidth access [28]. For such populations, it is crucial to support patients in the use of new technologies to ensure their successful implementation. Telepsychiatry interventions can be either self-directed (i.e. patient independently engages with the digital platform) or assisted (i.e. patient receives additional guidance from a professional). The former are potentially more scalable as they do not require a trained professional and can typically be delivered at lower cost. On the other hand, assisted telepsychiatry programs have been shown to lead to better patient outcomes than self-directed programs [29, 30] and will also be better suited for the populations mentioned above. Finally, there is substantial evidence on the cost-effectiveness of psychosocial interventions delivered by lay counsellors [31]. The assisted telepsychiatry model can combine the complementary strengths of task-sharing and digital technology to deliver exponential benefits in low-resource settings.

Our findings are consistent with evidence from various parts of the world that has demonstrated that telepsychiatry is broadly suitable for delivery of mental healthcare [13]. Telepsychiatry has been shown to be feasible in various settings, to deliver a variety of psychiatric treatments, in a range of ethnic groups and populations and across the lifespan [32]. Additionally, there is growing evidence demonstrating its effectiveness in delivering specific treatments and economic benefits through cost savings associated with reduced travel, improved care coordination and initiation of early treatment [32].

Most economic evaluations of telehealth indicate a net cost-saving to patients and the health system through several mechanisms [33]. These include reduced medical expenditure, avoidance of travel costs for the patients, caregivers and clinicians, reduced time off from work for patients and caregivers and reduced hospital stays and number of admissions/readmissions for chronic disease management [34–38]. These findings of significant savings in time and cost for patients receiving chronic disease care through telehealth have also been reported in LMICs [38]. While the initial setup costs telehealth might be high, there is sufficient research to suggest that long-term benefits far outweigh the initial investment [34, 39]. Finally. as technology costs keep decreasing, the telehealth costs per patient per year will also reduce, resulting in further cost-savings to the health system [40].

However, there are some key lessons from our study that we will need to pay attention to as technology becomes a key platform in delivery of services across the world. While some participants were comfortable with accessing healthcare over a digital platform, others did not find the experience optimal. While one would expect some of this discomfort to reduce with increased use of and familiarity with technology, the use of ‘assisted’ technology-enabled services, as we did, would help enhance acceptability. The focus of our program was to reduce supply-side barriers to access to mental healthcare. Although the placement of the service in primary healthcare allowed ease of access, demand-side barriers such as stigma continued to play a big role in influencing service uptake. Successful implementation of such programs will also need to consider components such as community awareness-building, which is designed to reduce demand barriers. Finally, while technology can serve to reduce geographical barriers to access, other key considerations for success include wider health systems reforms such as improved and consistent supply of medications.

The continuing disparities in the availability and inequitable distribution of mental health services between and within countries across the globe necessitates the development and expansion of innovative solutions such as telepsychiatry services. Although some barriers remain, the growing body of evidence supporting the feasibility and effectiveness of telepsychiatry and the lessons learnt from the COVID-19 pandemic, indicate that the time is ripe for adoption of such convenient, accessible and efficient approaches to increase access to mental healthcare. This will require training of healthcare providers on how to optimally deliver these services and development of systems for the storage and seamless sharing of electronic records. Furthermore, policymakers will need to invest in developing the necessary infrastructure as telepsychiatry has the potential to overcome the barriers related to shortage of specialist manpower in low-resource settings. While setting up telepsychiatry services will require front loading of costs for additional technology and use of bandwidth, these would be offset in the longer term by reduction in costs such as travel costs for patients and their families and by reduction in burden resulting from increased access to care. Obviously, greater cost savings would more likely be experienced by services with higher volumes of patients [41]. Finally, more robust studies and controlled evaluations are needed to explore socio-demographic predictors of feasibility and acceptability, diagnostic reliability, efficacy and cost-effectiveness of telepsychiatry in developing countries, as this model seems to be particularly suited for the specific needs and resources of the developing world.

Our study is one amongst the very few from LMICs that have explored the acceptability and feasibility of telepsychiatry integrated into routine healthcare services. When considered alongside the quantitative results [16], the qualitative evidence from the beneficiaries’ accounts helps to advance our understanding of the acceptability and feasibility of the program and recommendations for its scale up. This approach is essential for deployment of beneficiary-responsive services. We acknowledge several study limitations. Some of them are related to the inherent nature of qualitative research and include the limited generalizability due to the small sample size and the subjective nature of the analysis influenced by the positionality of the researchers. The other key limitation was that we did not select participants based on an improvement (or otherwise) and hence cannot discount the fact that their perceptions about the program might be influenced by their clinical status. Finally, it is important to note that none of these participants had previously been diagnosed with a mental health problem or received care from a mental health professional. Any comparison they might have made with face-to-face care would be based on their experience of general medical care for physical health problems, and this would also possibly have been influenced by their perception about the severity and urgency of their symptoms.

While there was substantial evidence about the effectiveness and acceptability of telepsychiatry [10], it was the COVID-19 pandemic that prompted swift and widespread uptake of technology for the preservation of doctor–patient interactions for ongoing therapeutic work [42]. Despite all its advantages, as we prepare health services in a post-pandemic world, we need to heed the lessons learnt while implementing telepsychiatry services during the pandemic. These include the limited suitability of such services for certain types of patients (e.g. those with diminished cognitive capacity or poor manual dexterity), the uneven distribution of access to technology, barriers to establishing rapport, inability to conduct a physical examination as a part of a comprehensive assessment and to pick on cues such as odour of alcohol, inability to respond to psychiatric emergencies and lack of certainty about privacy and confidentiality. Finally, it is important to note that several of these limitations can be possibly overcome through assisted telepsychiatry and this model could be further refined through lessons learnt from similar models that have been successfully deployed in other branches of medicine [43, 44].

## CONCLUSION

Assisted telepsychiatry when integrated in routine healthcare settings is perceived positively by service users and found to be acceptable and feasible to deliver. There is a community demand for the service to be adopted by the public health services. Psychoeducation, destigmatizing mental illness and better internet connectivity will help in maximizing its potential. This model should be adopted by public health services in low-resource settings to make scarce specialist services accessible despite geographical and logistical barriers. Finally, our findings are limited in their generalizability because of the small sample size and purposive nature of the sampling. However, this is consistent with the nature of qualitative studies where the focus is on the in-depth understanding of a phenomenon.
